# Medical Jurisprudence

**Published:** 1856-11

**Authors:** 


*Medical Jurisprudence.* By ALFRED S. TAYLOR, M.D. F.R.S. &c. Fourth American, from the Fifth and Improved London Edition, Edited, with Additions, by EDWARD HARTSHORNE, M.D. &c. Philadelphia, Blanchard & Lea, 1856.

This is one of those works which has found its way into the office of almost every physician in the country. It is only necessary to say, that this new edition possesses all the intrinsic value of former ones, together with much valuable matter with which the author has enriched the last London edition. It is for sale by Keen & Lee, Chicago. J.

